# The Variation in Chemical Composition and Source Apportionment of PM_2.5_ before, during, and after COVID-19 Restrictions in Zhengzhou, China

**DOI:** 10.3390/toxics12010081

**Published:** 2024-01-17

**Authors:** Jinting Huang, Aomeng Cai, Weisi Wang, Kuan He, Shuangshuang Zou, Qingxia Ma

**Affiliations:** 1 College of Surveying and Mapping Engineering, Yellow River Conservancy Technical Institute, Kaifeng 475004, China; jenkins1204@126.com; 2 Key Laboratory of Geospatial Technology for the Middle and Lower Yellow River Regions, Ministry of Education, College of Geography and Environmental Science, Henan University, Kaifeng 475004, China; 3 Henan Key Laboratory of Integrated Air Pollution Control and Ecological Security, Kaifeng 475004, China; 4 Henan Ecological and Environmental Monitoring Center, Zhengzhou 450007, China

**Keywords:** haze, reduced PM_2.5_ level, stable NO_3_^−^ level, high O_3_

## Abstract

Despite significant improvements in air quality during and after COVID-19 restrictions, haze continued to occur in Zhengzhou afterwards. This paper compares ionic compositions and sources of PM_2.5_ before (2019), during (2020), and after (2021) the restrictions to explore the reasons for the haze. The average concentration of PM_2.5_ decreased by 28.5% in 2020 and 27.9% in 2021, respectively, from 102.49 μg m^−3^ in 2019. The concentration of secondary inorganic aerosols (SIAs) was 51.87 μg m^−3^ in 2019, which decreased by 3.1% in 2020 and 12.8% in 2021. In contrast, the contributions of SIAs to PM_2.5_ increased from 50.61% (2019) to 68.6% (2020) and 61.2% (2021). SIAs contributed significantly to PM_2.5_ levels in 2020–2021. Despite a 22~62% decline in NO_x_ levels in 2020–2021, the increased O_3_ caused a similar NO_3_^−^ concentration (20.69~23.00 μg m^−3^) in 2020–2021 to that (22.93 μg m^−3^) in 2019, hindering PM_2.5_ reduction in Zhengzhou. Six PM_2.5_ sources, including secondary inorganic aerosols, industrial emissions, coal combustion, biomass burning, soil dust, and traffic emissions, were identified by the positive matrix factorization model in 2019–2021. Compared to 2019, the reduction in PM_2.5_ from the secondary aerosol source in 2020 and 2021 was small, and the contribution of secondary aerosol to PM_2.5_ increased by 13.32% in 2020 and 12.94% in 2021. In comparison, the primary emissions, including biomass burning, traffic, and dust, were reduced by 29.71% in 2020 and 27.7% in 2021. The results indicated that the secondary production did not significantly contribute to the PM_2.5_ decrease during and after the COVID-19 restrictions. Therefore, it is essential to understand the formation of secondary aerosols under high O_3_ and low precursor gases to mitigate air pollution in the future.

## 1. Introduction

Despite significant improvements in air quality since strict restrictions on residential and industrial activities were implemented to prevent the spread of COVID-19 [1,2,3,4,5,6], haze formed in the North China Plain (NCP). Many studies have reported that the efficient formation of secondary aerosol species, including nitrate and sulfate, induced occurrences of haze during the restrictions [7,8,9]. The formation of secondary aerosol species changed significantly before, during, and after the COVID-19 restrictions, but the factors of this variation remain unclear.

Nitrate (NO_3_^−^) and sulfate (SO_4_^2−^) are two main species of secondary inorganic aerosols that contributed to the haze pollution [10,11]. Their precursor gases (NO_x_ and SO_2_) are primarily produced by anthropogenic primary emissions. Due to COVID-19 restrictions, NO_x_ and SO_2_ concentrations have been reduced in the NCP. However, secondary pollution was enhanced in these anomalies, and they dominated the PM_2.5_ level [12,13,14].

SO_4_^2−^ is formed from SO_2_ through gas, aqueous, and heterogeneous reactions like those in the aqueous phase [11,12,15,16,17]. Studies found that the RH of ambient air was a major factor in SO_4_^2−^ conversion via heterogeneous reactions, and a high RH could enhance the heterogeneous formation of SO_4_^2−^ [18,19,20]. NO and NO_2_ are oxidized by a hydroxyl radical (OH) and O_3_ to produce nitric gas (HNO_3_), and then HNO_3_ is aerosolized with inorganic cations [21,22,23,24,25,26,27]. Wang et al. (2016) found that when NO_2_ levels are high, aerosol heterogeneous reactions from SO_2_ and NO_2_ produce SO_4_^2−^ and NO_3_^−^. In addition, the formation reactions of SO_4_^2−^ and NO_3_^−^ are influenced by NH_3_ and O_3_ concentrations, relative humidity (RH), and temperature [13,24,27,28].

COVID-19 restrictions, such as reduced transportation on roads, industry, construction sites, and restaurants, influenced anthropogenic pollutant emissions [29,30,31,32,33]. However, the large reduction (30~70%) in NO_x_ and decrease (15~30%) in PM_2.5_ did not eliminate the occurrence of haze [8,13,30,31,34]. Instead, O_3_ levels increased by 100~200% [32,35]. Studies have attempted to elucidate why haze events occurred during COVID-19 restrictions in China. Some papers confirmed that the contribution of SIAs to PM_2.5_ increased significantly in Beijing–Tianjin–Hebei and cities in northern China during restrictions, which prevented the continued decline in PM_2.5_, resulting in PM_2.5_ levels being higher than China’s National Ambient Air Quality Standards (NAAQS, PM_2.5_ of 75 μg m^−3^) [8,14,36,37,38,39]. It was reported that the enhanced contribution of sulfate and oxygenated organic aerosol inhibited further PM_2.5_ reduction in Shanghai, Lanzhou, and Xi’an [7,37,38]. Recent papers revealed that efficient secondary aerosol formation might have offset the reduction in primary emissions or decreased PM_2.5_ in Beijing and Hangzhou [8,9,38,40].

COVID-19 restrictions led to substantially reduced emissions of anthropogenic pollutants in big cities in the NCP. They provided an opportunity to investigate the response of air quality to reductions in air pollutants caused by human activities and the processes that caused the haze. Zhengzhou is one of the most populous cities in the southern NCP. Despite significant improvements in air quality in recent years, haze still occurs in Zhengzhou [41,42]. Restrictions were implemented in Zhengzhou (24 January to 18 February, 2020) to strictly regulate human, industrial, and construction activities to stop the spread of COVID-19. As the COVID-19 pandemic is still ongoing in Zhengzhou, changes in the chemical composition and source apportionment of PM_2.5_ and the reasons for these variations must be investigated in detail. This study compared the ionic composition and sources of PM_2.5_ in the city during restrictions with those before and after restrictions (24 January to 18 February, 2019 and 2021). This study analyzed the changes in NO_3_^−^ and SO_4_^2−^ formation before, during, and after restrictions and explored the causes of haze.

## 2. Methods

### 2.1. Observation Site and Instruments

**Figure 1:**
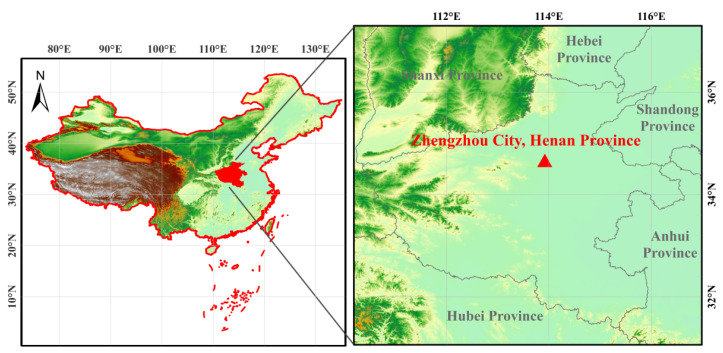
Location of the site (Zhengzhou) and topography in China.

The observation site was at the environment monitoring supersite of Henan province in Zhengzhou (34.76° N, 113.70° E, Figure 1). This study utilized observational data from 24 January to 18 February in 2019, 2020, and 2021. The three observation periods were studied and compared: 24 January to 18 February 2020 (the restriction period in Zhengzhou); 24 January to 18 February 2019 (the comparison period before the restriction period); and 24 January to 18 February 2021 (the comparison period after the restriction).

The meteorological conditions (pressure, temperature, relative humidity, wind speed, and wind direction) were measured by a weather station (WS601-UMB, Lufft Instruments, Inc., Stuttgart, Germany). The concentrations of PM_2.5_, PM_10_, O_3_, SO_2_, NO_x_, and CO were routinely measured through a series of pollutant detectors (5030, 5014i, 49I, 42I, 43I, 48I; Thermo Fisher Scientific, Waltham, MA, USA) at a time resolution of 5 min. A one-hour average of air pollutants and weather conditions was applied. 

An online analyzer for monitoring aerosols and gases (MARGA, model ADI 2080 Applikon Analytical B. V. Corp., Petten, The Netherlands) measured the hourly mass concentrations of NH_4_^+^, Na^+^, K^+^, Ca^2+^, Mg^2+^, SO_4_^2−^, NO_3_^−^, and Cl^−^ in PM_2.5_ at a flow rate of 16.7 L min^−1^ [43,44]. To ensure data accuracy, the seven-point calibration curve of each species and an internal standard solution were conducted before each campaign. In addition, sampling flow calibration and cyclone cleaning were conducted to eliminate instrumental drifts. The concentrations of organic carbon (OC) and elemental carbon (EC) in PM_2.5_ were measured using a thermo-optical OC/EC analyzer (Model RT-4, Sunset Lab, Tigard, OR, USA). Elements in PM_2.5_, including K, Ca, Zn, Cu Ni, Fe, Pb, Se, Cr, Mn, As, Co, Ti, Mo, Sc, V, Ba, Br, and Si, were quantified using an online analyzer for monitoring (AMMS-100, Focused Photonics Inc., Hangzhou, China). The formulas of major components in PM_2.5_ and the conversion rates of SO_2_ to sulfate (SOR) and NO_x_ to nitrate (NOR) are listed in Table 1. 

### 2.2. PMF Model 

The origins of PM_2.5_ were analyzed using the USEPA version 5.0 positive matrix factorization (PMF) model. This model is a widely used receptor model, has high efficiency and convenience without using pollution discharge conditions, and allows additional constraints to be added into the factor profiles or contributions to reduce results’ uncertainties. Input factors of the model include eight water-soluble ions (e.g., Na^+^, NH_4_^+^, K^+^, Mg^2+^, Ca^2+^, NO_3_^−^, SO_4_^2−^, and Cl^−^) and nine target metal elements (e.g., Si, Fe, Ti, Zn, Sb, Mn, Cu, Pb, and Cr), as well as the PM_2.5_ mass concentration. In this study, five source types were tested in the analysis. More details about PMF running and result diagnosis are in the Supplementary Materials.

## 3. Results 

### 3.1. Weather Conditions and Air Pollutants 

**Figure 2:**
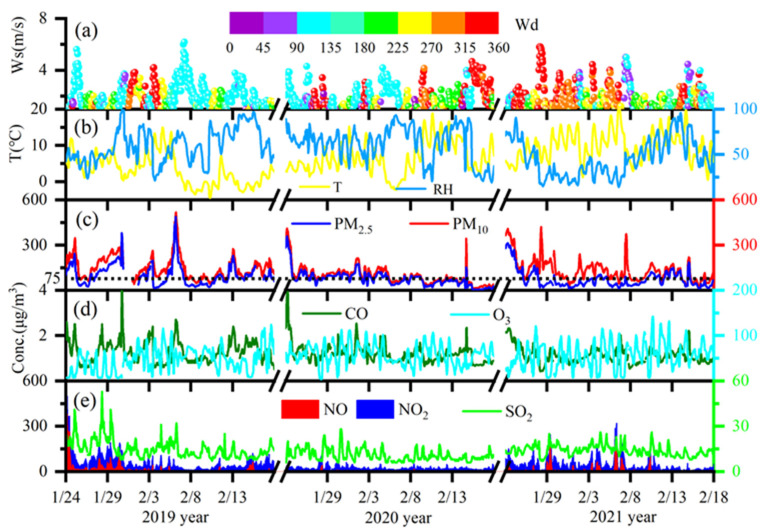
Evolution of meteorological conditions (**a**,**b**), particulate matter (PM_10_ and PM_2.5_) (**c**), and gaseous pollutants (NO, NO_2_, SO_2_, CO, and O_3_) (**d**,**e**) in 2019, 2020, and 2021.

Figure 2 illustrates the variations in wind direction (Wd), wind speed (Ws), relative humidity (RH), temperature (T), and air pollutant concentrations (PM_10_, PM_2.5_, CO, O_3_, NO_2_, NO, and SO_2_). The average wind speed (Wd) was 1.67 ± 1.01 m s^−1^ during the 2020 COVID-19 restrictions, 1.79 ± 1.06 m s^−1^ in 2019, and 1.67 ± 1.00 m s^−1^ in 2021. The results indicate that the wind was weak in all periods. The average temperature (T) was 3.20 ± 4.17 °C in 2019 and increased to 6.72 ± 4.41 °C in 2020 and 9.81 ± 3.99 °C in 2021. The average RH was 60–61% during the 2020 COVID-19 restrictions and in 2019, but 46% in 2021. 

**Figure 3:**
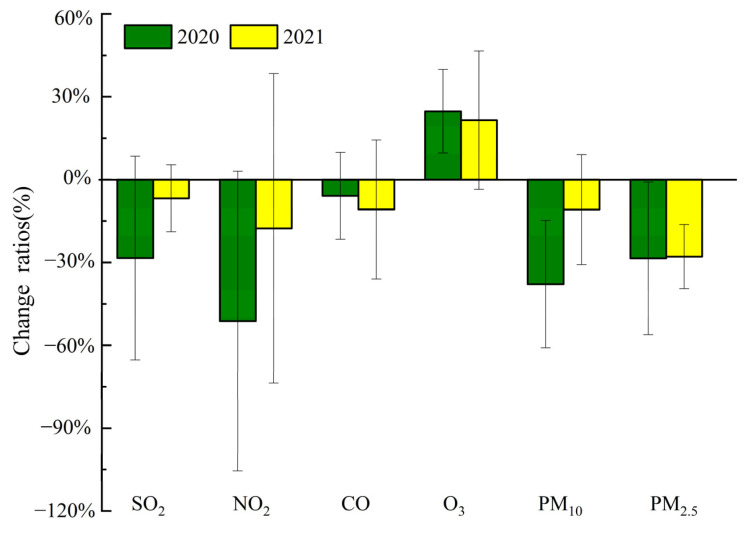
Change ratios of pollutant concentrations in 2020 and 2021 compared to 2019.

The average concentrations of PM_10_ and PM_2.5_ were 148.49 and 102.49 μg m^−3^ in 2019, and they decreased by 38% and 29% in 2020 and 11% and 28% in 2021 (Figure 3), respectively, resulting in a significant decline in particles in 2020 and 2021. However, the episodic haze formed with a PM_2.5_ value of more than 75 μg m^−3^ in 2020 and 2021. At the same time, the average concentrations of NO_2_, NO, SO_2_, and CO in 2019 were 44.45 ± 25.74 μg m^−3^, 24.89 ± 49.49 μg m^−3^, 14.90 ± 6.11 μg m^−3^, and 1.25 ± 0.49 mg m^−3^, respectively. NO_2_, NO, SO_2_, and CO concentrations decreased by approximately 51%, 90%, 28%, and 6% in 2020, respectively. In addition, NO_2_, NO, SO_2_, and CO concentrations in 2021 were lower by 18%, 58%, 7%, and 11% than in 2019. NO_2_, NO, SO_2_, and CO are mainly emitted by primary emissions. The reductions in NO_2_, NO, SO_2_, and CO levels indicated that primary emissions may have decreased in 2020 and 2021. In contrast, the concentration of O_3_ increased from 46.70 μg m^−3^ in 2019 to 58.24 μg m^−3^ in 2020 and 56.75 μg m^−3^ in 2021. The increased O_3_ concentration was found in many cities (e.g., Xi’an, Shanghai, Beijing, and Hangzhou) [7,9,39]. 

### 3.2. Chemical Compositions of PM_2.5_

The concentration of secondary inorganic aerosols (SIAs) was 51.87 μg m^−3^ in 2019, 50.25 μg m^−3^ during the 2020 COVID-19 restrictions, and 45.21 μg m^−3^ in 2021. Compared with that in 2019, the SIA concentration decreased by 3.1% in 2020 and 12.8% in 2021. In contrast, the contributions of SIAs to PM_2.5_ increased from 50.61% in 2019 to 68.6% in 2020 and 61.2% in 2021. Between 2019 and 2021, SIAs were the major components of PM_2.5_, with a greater impact in 2020 and 2021. Compared with that in 2019, the concentration of PM_2.5_ decreased by 29% during the 2020 COVID-19 restrictions and 28% in 2021, but the SIA concentration decreased by 3% and 13%, respectively. The different declines between PM_2.5_ and their SIAs may be attributed to NO_3_^−^ and SO_4_^2−^ formation. 

**Figure 4:**
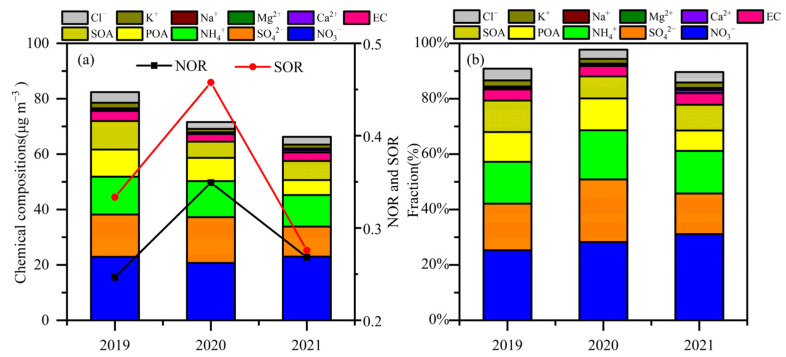
Average mass concentrations (**a**) and mass fractions (**b**) of PM_2.5_ constituents (SO_4_^2−^, NO_3_^−^, NH_4_^+^, POA, SOA, EC, Cl^−^, K^+^, Na^+^, Ma^2+^, and Ca^2+^), NOR, and SOR in 2019, 2020, and 2021.

The average concentrations of NO_3_^−^, NH_4_^+^, and SO_4_^2−^ were 22.93 μg m^−3^, 13.69 μg m^−3^, and 15.25 μg m^−3^ in 2019 (Figure 4a). The average NO_3_^−^ and NH_4_^+^ concentrations were reduced by 9.8% and 5%, respectively, during the 2020 restriction period. In contrast, SO_4_^2−^ increased by 8.6% in 2020. These results indicate that the reduction in precursor gases (NO_x_ and SO_2_) did not result in proportionate reductions in NO_3_^−^ and SO_4_^2−^ during the 2020 restriction period compared to 2019. Mismatched decreases in NO_3_^−^ levels with marked NO_x_ reductions and the puzzling SO_4_^2−^ increase during the 2020 restriction period offset the decrease in PM_2.5_ levels in Zhengzhou. The average concentrations of NO_3_^−^ in 2021 were similar to that in 2019. However, the average NH_4_^+^ and SO_4_^2−^ concentrations decreased by 16.9% and 28.9%, respectively, in 2021. The large reductions (18% and 58%) in NO_2_ and NO also could not help alleviate NO_3_^−^ formation in 2021, which was related to the high conversion rate of NO_x_ to nitrate (NOR). The restriction resulted in a decrease in PM_2.5_ levels; however, air quality remained episodically bad.

The average concentrations of OC and EC were 12.71 and 3.64 μg m^−3^ in 2019, accounting for 12% and 4% of PM_2.5_ mass (Figure 4b). The average concentrations of OC and EC were 8.97 and 2.76 μg m^−3^ in 2020, 29% and 24% lower than those in 2019, respectively. In 2021, the average concentrations of OC and EC decreased by 38% and 15%, respectively. Although the average concentrations of OC and EC showed significant reductions in 2020 and 2021, the contributions to PM_2.5_ were similar. The value of OC/EC was 4.31 (1.83~11.93) in 2019, 3.64 (1.99~9.71) in 2020, and 2.77 (1.16~8.74) in 2021. OC and EC are majorly emitted from fossil fuel combustion and biomass burning. Carbonaceous aerosols from biomass-burning sources have richer OC than EC, implying high OC/EC values. When OC/EC ratios were lower than 2.0, carbonaceous aerosols were from vehicular and industrial emissions. In addition, high OC/EC ratios (>2.0) indicate the presence of secondary organic aerosols (SOAs) [7,37,43]. OC/EC ratios mostly exceeded 2 in 2019 and 2021 (Figure S1), suggesting carbonaceous aerosols from biomass-burning sources. The mass concentration of SOA decreased from 10.31 μg m^−3^ in 2019 to 5.83 μg m^−3^ in 2020 and 6.88 μg m^−3^ in 2021 (Figure 4 and Table 1). The reductions in SOA were 43.49% in 2020 and 33.21% in 2021. The changes in secondary inorganic species were much smaller than those in secondary organic aerosols.

The average concentrations of Cl^−^ and K^+^ decreased from 2.03 and 3.84 μg m^−3^ in 2019 to 1.25 and 2.45 μg m^−3^ in 2020 and to 1.47 and 2.79 μg m^−3^ in 2021, implying a reduction in emissions from coal combustion and biomass burning in 2020 and 2021. The average concentrations of Ca^2+^, Mg^2+^, and Na^+^ in 2019 were 0.46, 0.19, and 0.29 μg m^−3^, respectively, which were 40%, 31%, and 26% higher than those in 2020 and 93%, −2%, and 7% lower than those in 2021.

### 3.3. Nitrate and Sulfate Formations over the Three Years 

It is a puzzling phenomenon that SIA contributions to PM_2.5_ were higher in 2020 (~68%) and in 2021 (~60%) than in 2019 (51%), when PM_2.5_ decreased from 102.49 μg m^−3^ (2019) to 73.92 μg m^−3^ (2020) and 76.78 μg m^−3^ (2021). SO_2_ and NO_x_ are NO_3_^−^ and SO_4_^2−^’s key precursor gases. To elucidate the puzzling phenomenon, the formation of NO_3_^−^ and SO_4_^2−^ was compared with their precursor gases, PM_2.5_, and conversion ratios under different periods. 

**Figure 5:**
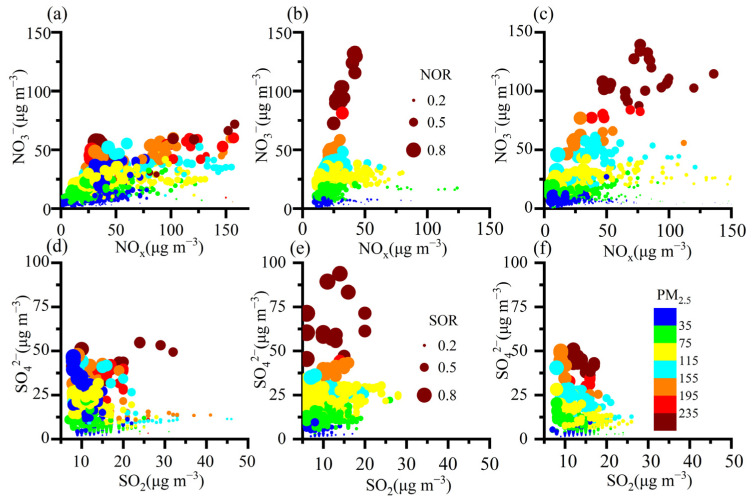
Correlations between NO_3_^−^, SO_4_^2−^, and precursor gases in 2019 (**a**,**d**), 2020 (**b**,**e**), and 2021 (**c**,**f**). Symbols in (**a**–**f**) are scaled by conversion ratios (NOR and SOR) and colored by PM_2.5_ concentrations.

NO_3_^−^ positively increased with NO_x_ between 2019 and 2021 (Figure 5 and Table S2, *p* < 0.01), indicating that high concentrations of the precursor contributed to high NO_3_^−^ levels and then led to the remarkable increases in PM_2.5_. The result was supported by the positive relationship between NO_3_^−^ and PM_2.5_ (Table S2, *p* < 0.01). At a given precursor concentration, higher conversion ratios led to higher NO_3_^−^ and SO_4_^2−^ concentrations, implying that secondary conversion ratios were important for the formation of NO_3_^−^ and SO_4_^2−^. It was found that NO_3_^−^ and SO_4_^2−^ levels were low in the region with high precursor gases region and low conversion ratios. The results suggested that the absolute concentrations of NO_3_^−^ and SO_4_^2−^ were more closely related to the secondary conversion rates than the precursor concentrations. A similar phenomenon was found in the summer of 2019 and 2020 [3].

There were some exceptions in the same NO_x_ region when NO_3_^−^ concentrations were higher in 2020 and 2021 than in 2019. In 2020 and 2021, the higher NO_3_^−^ levels in the same NO_x_ region were closely related to the higher NOR. Meanwhile, in 2020, higher SO_4_^2−^ concentrations in the same SO_2_ region significantly correlated with higher SOR levels. It is equally inconceivable that higher conversion ratios occurred in 2020 and 2021 to cause high secondary formation, especially NO_3_^−^.

**Figure 6:**
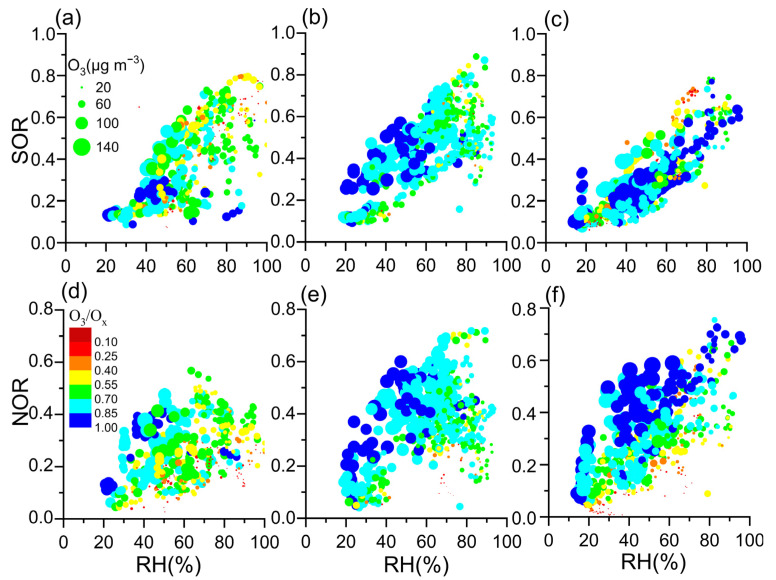
Correlations between conversion ratios (NOR and SOR) and RH in 2019 (**a**,**d**), 2020 (**b**,**e**), and 2021 (**c**,**f**). Symbols in (**a**–**f**) are scaled by O_3_ concentration and colored by O_3_/O_x_. The oxidants (O_x_) are the sum of NO_2_ and O_3_, a proxy for atmospheric oxidation caused by photochemical reactions.

RH and O_3_ are key factors regulating the oxidation pathways for nitrate and sulfate formation [14,15,37,45]. Both NOR and SOR were positively correlated with RH, as shown in Figure 6 and Table S4 (*p* < 0.01), suggesting that high RH favors the conversion of gaseous precursors (e.g., NO_x_ and SO_2_) to NO_3_^−^ and SO_4_^2−^. The NOR concentration was high in a high-O_3_ region at a given RH, while the SOR was not (Figure 6). NOR was positively correlated with O_3_ and O_3_/O_x_ in 2019–2021 (*p* < 0.01,Table S4), but SOR was not. The results indicated that NO_x_ was efficiently oxidized by O_3_. O_3_ increased in 2020 and 2021; higher NOR values were accompanied by higher O_3_ and O_3_/O_x_ values in a given RH, compared with 2019 (Figure 6), whereas the conversion rate of SO_2_ to sulfate was not. O_3_ was likely the major oxidant for the high level of NO_3_^−^ formation. 

### 3.4. Sources during the Three Periods

**Figure 7:**
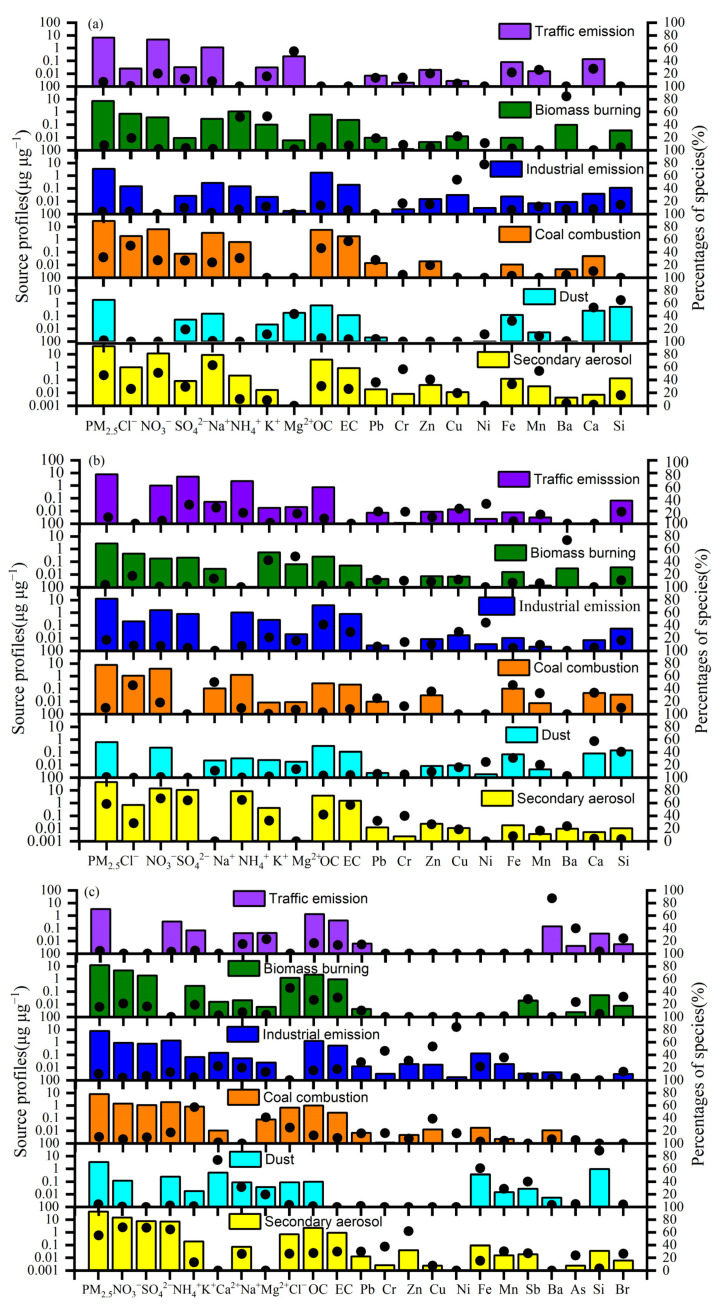
Six sources’ profiles (bars) (in units of μg μg^−1^) and contribution percentages (black dots) from each source factor resolved from the PMF model in 2019 (**a**), 2020 (**b**), and 2021 (**c**).

The profiles of six factors were identified using the EPA PMF v5.0 in Figure 7. Factor 1 was characterized by the highly explained variations in Ni, Zn, Se, Mg^2+^, Fe, and Mn in the three periods, identified as traffic emissions. Zn is widely used as an additive for lubricants in engines, vehicular tailpipe exhausts [45], debris from brake wear, and worn tires [46,47]. Factor 2 was dominated by high K^+^, Ba, and Mg^2+^ concentrations, generally indicators of biomass burning [48,49]. Factor 3 was characterized by highly explained variations in Ni, As, Cu, Ni, Mo, OC, and EC in 2019 and 2020, whereas Ba, As, OC, and EC in 2021 were identified as industrial emissions. This result was consistent with different industrial processes in the three areas. Studies have shown that steel sintering can emit large amounts of As, Zn, Ni, and Cu [21,50]. Factor 4 was dominated by high Cl^−^, OC, and EC concentrations, generally indicators of coal combustion [21,40,51]. Factor 5 included large amounts of Mg^2+^, Ca^2+^, and Si [52,53,54]. Previous studies have shown that soil is also represented by a high loading of Ca, Mg, Si, and Al [47,49,54]; therefore, this factor was attributed to dust. Factor 6 was characterized by high NO_3_^−^, SO_4_^2−^, NH_4_^+^, and OC content, as well as relatively high concentrations of OC. These species are mainly associated with secondary processes [46,50]. Thus, this factor was classified as a secondary aerosol.

Secondary processes (43.09 μg m^−3^), coal combustion (29.18 μg m^−3^), biomass burning (7.25 μg m^−3^), traffic emissions (6.59 μg m^−3^), industrial emissions (3.48 μg m^−3^), and dust (1.87 μg m^−3^) were the six main sources of PM_2.5_ in Zhengzhou in 2019, contributing 42.04%, 28.47%, 7.08%, 6.43%, 3.39%, and 1.82% of the PM_2.5_, respectively (Table S5). Compared with 2019, the PM_2.5_ mass concentration from the secondary aerosols in 2020 was similar to that in 2019, but the contribution of this source to PM_2.5_ increased by 13.32%, which was consistent with the secondary inorganic components. In 2020, the PM_2.5_ mass concentrations emitted by coal combustion, biomass burning, traffic emissions, industrial emissions, and dust showed obvious decreases. However, the contribution of coal combustion to PM_2.5_ mass concentrations slightly increased by 2.87%, and the contributions of other sources to PM_2.5_ mass concentrations slightly decreased by 0.41~3.27%. These results indicate that the changes in primary emissions from coal combustion, biomass burning, traffic emissions, industrial emissions, and dust were similar to those in the PM_2.5_ level in 2020. The PM_2.5_ mass concentrations from secondary aerosols, coal combustion, and traffic emissions in 2021 were lower than those in 2019, but the PM_2.5_ mass concentrations emitted by industrial emissions and dust were higher. In 2021, the contribution of secondary aerosols to PM_2.5_ levels was higher by 12.92% than that in 2019. However, coal combustion’s contribution declined by 12.69%, suggesting that primary emissions from coal combustion were significantly reduced in 2021, while the influence of the secondary process on the PM_2.5_ decreases was small. Secondary aerosols were the main source of PM_2.5_ between 2019 and 2021, playing the more dominant role in PM_2.5_ levels in 2020 and 2021 despite substantial reductions in gas emissions in 2020 and 2021. The primary emissions (biomass burning, traffic, and dust) were reduced by 29.71% in 2020 and 27.7% in 2021 compared to 2019. However, the PM_2.5_ concentrations from the secondary processes in 2020 and 2021 were slightly decreased, and the contribution of the secondary processes to PM_2.5_ increased by 13.32% in 2020 and 12.94% in 2021. The reductions in PM_2.5_ levels in 2020 and 2021 were mainly due to a decline in primary emissions rather than the secondary process. A similar scenario, with higher contributions from secondary pollution to unexpected PM_2.5_ levels, was found in many cities with COVID-19 restrictions [28,38,51,52,53,54].

## 4. Discussion

Significant improvements in air quality occurred during and after the COVID-19 outbreak in China, but haze still formed [1,2,13,34,41,55]. Many studies have found that enhancing secondary pollution inhibits these anomalies’ continuous decline in PM_2.5_ levels [9,32,38,56]. Changes in secondary aerosol formation under low precursor gas (NO_x_ and SO_2_) levels with high O_3_ levels throughout this pandemic remain unclear. Due to restrictions on residential and industrial activities implemented to prevent the spread of COVID-19 in Zhengzhou, PM_2.5_ concentrations showed a remarkable decline of 29% during the 2020 COVID-19 restrictions compared with 2019. In addition, after the COVID-19 restrictions were lifted, PM_2.5_ concentrations in 2021 were 28% lower than in 2019 (Figure 3). Moreover, the average molar concentration of total nitrogen compounds (NO_x_ + NO_3_^−^) in the air decreased from 1.60 µmol m^−3^ in 2019 to 0.89 µmol m^−3^ in 2020 and 1.44 µmol m^−3^ in 2021. Meanwhile, that of sulfur compounds (SO_2_ + SO_4_^2−^) also slightly decreased from 0.39 µmol m^−3^ (2019) to 0.34 µmol m^−3^ (2020) and 0.33 µmol m^−3^ (2021), indicating that the reduction in nitrogen compounds during the 2020 COVID-19 restrictions was larger than that in 2021, while the decline in sulfur compounds was small. Ma et al. (2022) found that the average molar concentration of total nitrogen compounds before COVID-19 restrictions (January 1 to 23, 2020) was 2.74 µmol m^−3^, obviously higher than that during the COVID-19 restrictions; meanwhile, that of sulfur compounds (0.36 µmol m^−3^) was slightly higher than that during the COVID-19 restrictions.

Despite significant decreases in total nitrogen in 2020 and 2021, NO_3_^−^ levels were similar to that in 2019, while NO and NO_2_ levels were significantly lower than that in 2019. This result indicates that nitrogen levels significantly changed when partitioning into NO_3_^−^, NO_2_, and NO in 2020 and 2021. It is a puzzle why a higher distribution of NO_3_^−^ occurred with lower NO and NO_2_ in 2020 and 2021 than in 2019. It was found that the O_3_ concentration was 1.21 μmol m^−3^ in 2020 and 1.18 μmol m^−3^ in 2021, higher than 0.97 μmol m^−3^ in 2019. O_3_ molar concentrations in 2020 and 2021 were far greater than NO_2_ molar concentrations (0.47 μmol m^−3^ and 0.80 μmol m^−3^), respectively, implying that high O_3_ promoted the conversion of NO to NO_2_, then to NO_3_, and finally to NO_3_^−^. Thus, NO levels in 2020 and 2021 were obviously lower than in 2019 (Table S1). The high production of NO_3_^−^ from NO_x_ could have offset the effect of reduced total nitrogen on PM_2.5_ levels [1,2].

**Figure 8:**
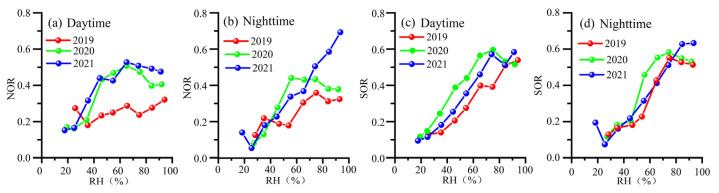
NOR and SOR with RH in the daytime (08:00–19:00) (**a**,**c**) and nighttime (20:00–07:00) (**b**,**d**) in 2019 2020 and 2021.

NOR positively correlated with RH in 2019–2021 (Figure 6a,b and Table S4, *p* < 0.01). NOR was much higher in 2020 and 2021 than in 2019, when RH was higher than 40%. This result is consistent with high RH favoring NO_3_^−^ formation via NO_x_ in 2020 and 2021 (Figure 6, Table S4). The increase in NOR with RH in the daytime was significantly higher than at nighttime (Figure 8). Moreover, the variations in NOR, O_3_/O_x_, and O_3_ concentration were similar in the daytime (high) and nighttime (low) in 2019–2021 (Figure S4). Additionally, high O_3_/O_x_ and O_3_ concentrations in the daytime in 2020–2021 were accompanied by high NOR. In 2020 and 2021, high NOR meant high NO_3_^−^ formation from NO_x_. As shown in Figure 6, the high NOR was positively related to high O_3_ and O_3_/O_x_ (Table S4). The formation was likely because high O_3_ promoted the conversion of NO to NO_2_, resulting in more NO_2_ to form NO_3_^−^. If O_3_ concentrations in 2020 and 2021 were similar to those in 2019, the NOR might have been similar to that (0.25) in 2019. NO_3_^−^ concentrations in 2020 decreased from 20.36 μg m^−3^ to 11.44 μg m^−3^ in 2020, while NH_4_^+^ levels fell by 3.32 μg m^−3^. Thus, PM_2.5_ levels decreased from 73.24 to 61.00 μg m^−3^ during the COVID-19 restriction period. Similarly, NO_3_^−^ and NH_4_^+^ declined by 5.16 μg m^−3^ and 1.49 μg m^−3^ in 2021, resulting in a decrease (~7 μg m^−3^) in PM_2.5_ levels. Enhanced NOR hindered a ~17% and ~10% decline in PM_2.5_ levels during and after the COVID-19 restrictions in Zhengzhou. As a result of the increased O_3_, NO_3_^−^ formation was enhanced by oxidizing NO to NO_2_, offsetting the reduction in PM_2.5_ levels.

SOR increased with RH between 2019 and 2021 (Figure 6c,d). This result indicates that moist air favors the conversion of SO_2_ to SO_4_^2−^. Moreover, the variation in SOR with RH was similar in the daytime and nighttime. O_3_ concentration was high in the daytime and low at nighttime (Figures S4 and S5). On the contrary, SOR and RH levels were low in the daytime and high at nighttime. Figure 6a–c show that high SOR did not follow high O_3_ levels under certain RH values between 2019 and 2021. Figure S3 and Table S4 depict the poor correlation between SOR and O_3_. The results suggest that photochemical reactions were not the major pathway of the SO_4_^2−^ formation. Thus, the heterogeneous reactions on the particle surface were the major route for SO_4_^2−^ formation, rather than photochemical reactions.

As shown in Figure S2, the slopes of NH_4_^+^ and NO_3_^−^ + 2 SO_4_^2−^ were 1.11 in 2019, 1.02 in 2020, and 1.02 in 2021, implying that NH_4_^+^ was rich in 2019. The enhanced formation of nitrate and sulfate in 2020 and 2021 may not have been caused by rich NH_4_^+^. The wind speed was generally less than 2 m s^−1^ in 2019–2021 (Figure S6). Temperature has negative correlations with NO_3_^−^, SO_4_^2−^, and conversion ratios (NOR and SOR) (Tables S2–S4). The NO_3_^−^ had similar levels in 2019–2021, despite lower total nitrogen compounds in 2020 and 2021 than in 2019. Thus, wind speed and temperature were not major factors for the enhanced NO_3_^−^. The normalized concentrations of NO_3_^−^ by EC were to counteract the effect of the atmospheric physical processes and represent the contribution from chemical reactions [56] because EC was produced only from primary emissions and was a very inertial species to the chemical reactions. Its variability was mainly controlled by emission intensity and atmospheric physical processes. The NO_3_^−^/EC ratio increased from 6.30 in 2019 to 7.50 in 2020 and 7.44 in 2021, reflecting an elevated production of NO_3_^−^. The results were consistent with the variations in NO_3_^−^ contributions to PM_2.5_. An earlier study also observed a similar phenomenon [38,52,53,54]. Another earlier study reported that NOR increased from 0.14 to 0.21 in 2020 when Shanghai implemented restrictions [1]. In Xi’an, NOR increased from 0.33 in non-restriction periods to 0.57 in restriction periods, when O_3_ concentration increased from 35.3 to 53.6 μg m^−3^ [38]. An investigation of the published data on haze occurrence during and after COVID-19 restrictions in many cities in China revealed a similar scenario, with photochemically enhanced NOR causing major contributions of NO_3_^−^ to PM_2.5_ and inhibiting the continuous decline in PM_2.5_ levels [1,9,25,38].

## 5. Conclusions

Despite significant improvements in air quality during and after COVID-19 restrictions, due to strict regulations on residential and industrial activities to curb the spread of COVID-19, haze still occurred in Zhengzhou. The average concentration of PM_2.5_ decreased from 102.49 μg m^−3^ before the restrictions (2019) to 73.24 μg m^−3^ during the restrictions (2020) and 73.90 μg m^−3^ after the restrictions (2021). In 2019, SIA concentrations declined by 3.1% in 2020 and 12.8% in 2021. At the same time, the contributions of SIAs to PM_2.5_ increased to 68.6% (2020) and 61.2% (2021), higher than 50.61% in 2019. SIAs caused major contributions to PM_2.5_ levels in 2020 and 2021. NO_x_ levels in 2020 and 2021 were 22~62% lower than that in 2019; however, NO_3_^−^ concentrations had similar levels (20.69~23.00 μg m^−3^) in 2019, 2020, and 2021, which was attributed to the enhanced conversion rate of NO_x_ to nitrate by the increased O_3_ levels in 2020 and 2021. The inconsistent reduction between nitrate and NO_x_ inhibited further PM_2.5_ reduction and contributed to haze formation in Zhengzhou.

Six of the PM_2.5_ sources were secondary inorganic aerosols, industrial emissions, coal combustion, biomass burning, soil dust, and traffic emissions in 2019, 2020, and 2021. Compared with 2019, the primary emissions (biomass burning, traffic, and dust) were reduced by 29.71% in 2020 and 27.7% in 2021. However, the PM_2.5_ from the secondary process in 2020 and 2021 decreased slightly, and the contribution of the secondary process to PM_2.5_ increased by 12.94%~13.32% in 2020 and 2021. The reductions in PM_2.5_ levels in 2020 and 2021 were mainly due to a decline in primary emissions rather than the secondary process. Thus, a better understanding of the formation of secondary aerosols under high O_3_ and low precursor gases would be needed to help mitigate air pollution in the future.

## Figures and Tables

**Table 1:** Formulas and references of main components in PM_2.5_.

Main Components	Formulas	References
Secondary inorganic aerosols (SIAs)	SO_4_^2−^ + NO_3_^−^ + NH_4_^+^	[27]
Primary organic aerosol (POA)	ρ[POA] = 1.6 × ρ[EC] × (ρOC/ρEC)min	[36]
Secondary organic aerosol (SOA)	ρ[SOA] = 1.6×(ρ[OC] − ρ[POC])	[43]
The conversion ratios of SO_2_ to sulfate (SOR)	[SO_4_^2−^]/([SO_4_^2−^] + [SO_2_])	[37]
The conversion ratios of NO_x_ to nitrate (NOR)	[NO_3_^−^]/([NO_3_^−^] + [NO] + [NO_2_])	[13]

Where ρ[x] is the mass concentration of x species (μg m^−3^). [x] is the molar concentration of x species (μmol m^−3^).

## Data Availability

The data presented in this study are available on request from the corresponding author.

## Supplementary Materials

The following supporting information can be downloaded at: https://www.mdpi.com/article/10.3390/toxics12010081/s1. Figure S1: The OC/EC ratios in 2019 (a), 2020 (b) and 2021 (c); Figure S2: NOR and SOR with O_3_ in 2019, 2020 and 2021; Figure S3: NOR and SOR with O_3_ in 2019, 2020 and 2021; Figure S4: The diurnal variations of NOR and SOR in 2019 (a), 2020 (b) and 2021 (c); Figure S5: The diurnal variations of RH, O_3_ and O_3_/O_x_ in 2019 (a), 2020 (b) and 2021 (c); Figure S6: Statistical analysis of wind speed in 2019–2021; Table S1: Average mass concentrations (µg m^−3^) of air pollutants and species in PM_2.5_ in 2019, 2020 and 2021; Table S2: Correlation analysis between NO_3_^−^, PM_2.5_, T, RH and NOR in 2019–2021 (Pearson correlation coefficients); Table S3: Correlation analysis between SO_4_^2−^, PM_2.5_, T, RH and SOR in 2019-2021 (Pearson correlation coefficients); Table S4: Correlation analysis between conversion ratios (NOR and SOR), RH, O_3_ and O_3_/O_x_ in 2019–2021 (Pearson correlation coefficients); Table S5: Average contributions (%) of six sources to PM_2.5_ in 2019, 2020 and 2021.
