# Supplementary material for: The Variation in Chemical Composition and Source Apportionment of PM2.5 before, during, and after COVID-19 Restrictions in Zhengzhou, China
Source: Toxics. 2024 Jan 17;12(1):81. doi: 10.3390/toxics12010081 (PMC10819188; doi:10.3390/toxics12010081)
Supplement: Supplementary file 1 [file toxics-12-00081-s001.zip › toxics-2785921-supplementary.pdf]

toxics MDPI

Supplementary Materials: The Variation in Chemical Composition and Source Apportionment of $PM_{2.5}$ before, during, and after COVID-19 Restrictions in Zhengzhou, China

Jinting Huang, Aomeng Cai, Weisi Wang, Kuan He, Shuangshuang Zou and Qingxia Ma

Positive matrix factorization (PMF)

Positive matrix factor analysis model (US EPA PMF 5.0) was used to analyze the sources of chemical components in $PM_{2.5}$. PMF is a multivariate receptor model, assuming that the concentration of $PM_{2.5}$ components at the receptor is a linear combination of pollution source contributions. Equation based uncertainty includes detection limits and error scores. If the concentration is less than or equal to the detection limit (MDL), the uncertainty (UNC) is calculated using the following formula:

$$UNC=5/6 \times MDL$$

If the concentration is greater than MDL, the formula is:

$$UNC=\sqrt{(ErrorFraction \times concentration)^2 + (0.5 \times MDL)^2}$$

Qtrue/Qexp is an indicator of the optimal solution. To find the optimal factor number, it was tested from 3 to 7. if the Qtrue/Qexp number approaches 1, indicating the optimal factor number is properly estimated. The Qtrue/Qexp approached 1 for the six factor solution in this study. In addition, the error fraction in the PMF analysis was set to 10% for the elements. More details about PMF running and result diagnosis can be found elsewhere (Zheng et al., 2020) .

Zheng, H., Kong, S., Chen, N., Yan, Y., Liu, D., Zhu, B., Xu, K., Cao, W., Ding, Q., Lan, B., Zhang,Z., Zheng, M., Fan, Z., Cheng, Y., Zheng, S., Yao, L., Bai, Y., Zhao, T., Qi, S. Significant changes in the chemical compositions and sources of PM2.5 in Wuhan since the city lockdown as Covid-19. Sci. Total Environ 2020, 739, 140000.31.

Table S1. Average mass concentrations (μg m^{-3}) of air pollutants and species in $PM_{2.5}$ in 2019, 2020 and 2021.

Species	2019	2020	2021
$PM_{2.5}$	102.49 ± 70.33	73.24 ± 50.88	73.90 ± 57.56
PM_{10}	148.49±76.95	92.26±59.21	132.34±73.92
SO_2	14.90±6.11	10.67±3.86	13.89±3.44
NO_x	67.29±80.47	25.32±14.58	52.45±55.72
NO	24.89±49.49	2.47±3.03	10.44±23.69
O_3	46.70±26.27	58.24±22.30	56.75±29.74
NO_3^-	22.93 ± 15.14	20.69 ± 17.60	23.00 ± 24.23
NH_4^+	13.69 ± 8.86	13.00 ± 9.67	11.37 ± 11.03
SO_4^{2-}	15.25 ± 11.40	16.56 ± 12.08	10.84 ± 10.68
K^+	2.03 ± 3.78	1.25 ± 1.22	1.47 ± 2.15
Cl^-	3.84 ± 3.95	2.45 ± 1.89	2.79 ± 2.01
OC	12.71 ± 7.42	8.97 ± 4.50	7.93 ± 4.46
EC	3.64 ± 2.77	2.76 ± 1.85	3.09 ± 1.75
SOR	0.33±0.23	0.46±0.20	0.28±0.20
NOR	0.25±0.17	0.35±0.18	0.27±0.19

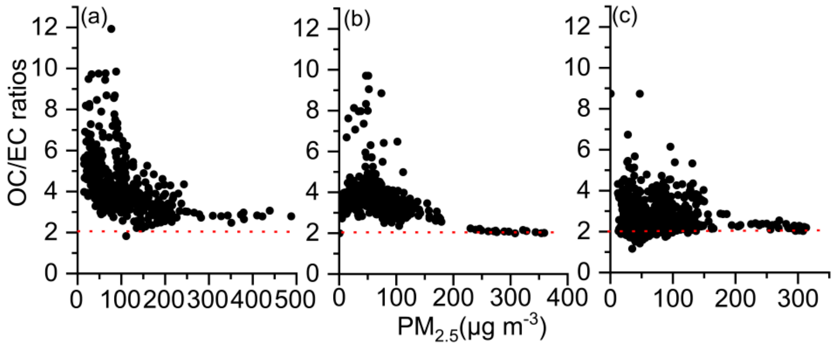

Figure S1. The OC/EC ratios in 2019 (a), 2020 (b) and 2021(c).

Table S2. Correlation analysis between NO_3^-, $PM_{2.5}$, T, RH and NOR in 2019-2021(Pearson correlation coefficients)

	NO_3^-		
	2019	2020	2021
$PM_{2.5}$	0.828*	0.931*	0.959*
T	-0.041	-0.177*	-0.311*
RH	0.346*	0.314*	0.472*
NOR	0.135*	0.684*	0.480*

(*： p<0.01)

Table S3. Correlation analysis between SO_4^{2-}, $PM_{2.5}$, T, RH and SOR in 2019-2021(Pearson correlation coefficients)

	SO_4^{2-}		
	2019	2020	2021
$PM_{2.5}$	0.750*	0.896*	0.931*
T	-0.143*	-0.215*	-0.282*
RH	0.602*	0.376*	0.674*
SOR	0.873*	0.740*	0.847*

(*： p<0.01)

Table S4. Correlation analysis between conversion ratios (NOR and SOR), RH, O_3 and O_3/O_x in 2019-2021(Pearson correlation coefficients)

	NOR			SOR		
	2019	2020	2021	2019	2020	2021
$PM_{2.5}$	-0.030	0.709*	0.501*	0.269*	0.669*	0.744*
RH	0.200*	0.457*	0.659*	0.626*	0.707*	0.803*
T	-0.367*	-0.243*	-0.048	-0.050	-0.379*	-0.239*
O_3	0.312*	0.161*	0.379*	-0.016	-0.279*	-0.045
O_3/O_x	0.465*	0.217*	0.450*	-0.012	-0.298*	0.058

(*： p<0.01)

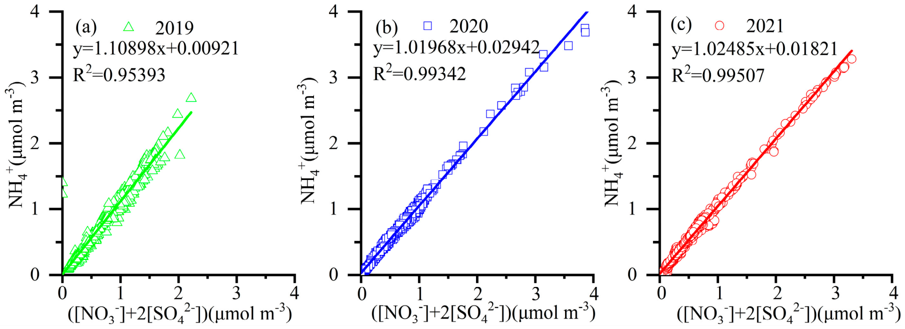

Figure S2. NOR and SOR with O_3 in 2019, 2020 and 2021.

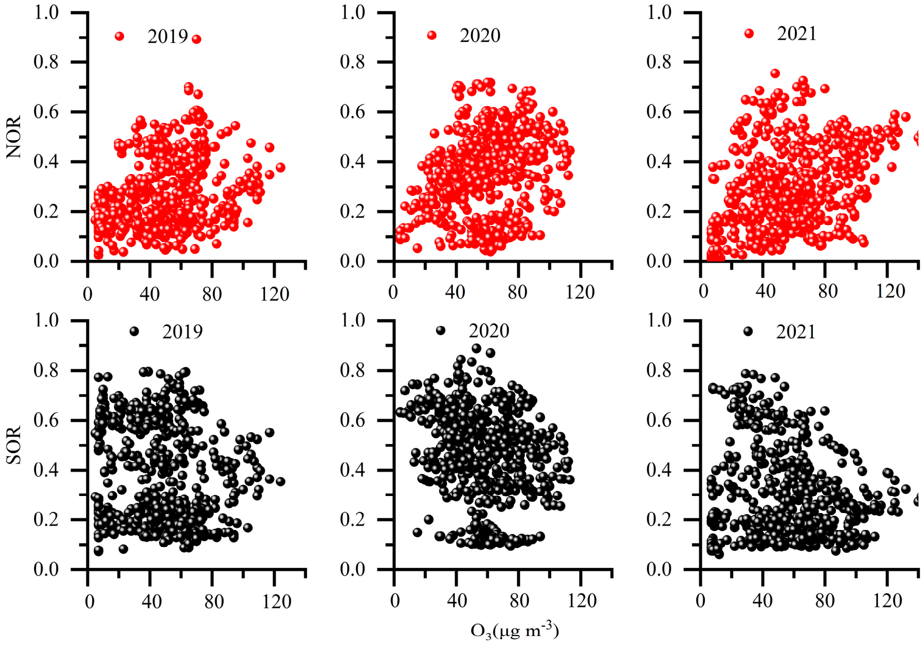

Figure S3. NOR and SOR with O_3 in 2019, 2020 and 2021.

Table S5. Average contributions (%) of six sources to $PM_{2.5}$ in 2019, 2020 and 2021.

Sources	2019	2020	2021
Secondary process	42.04	55.36	54.98
Coal combustion	28.47	31.34	15.78
Biomass burning	7.08	3.81	10.39
Traffic emission	6.43	4.88	4.34
Industrial emission	3.39	2.37	10.20
Dust	1.82	2.23	4.32

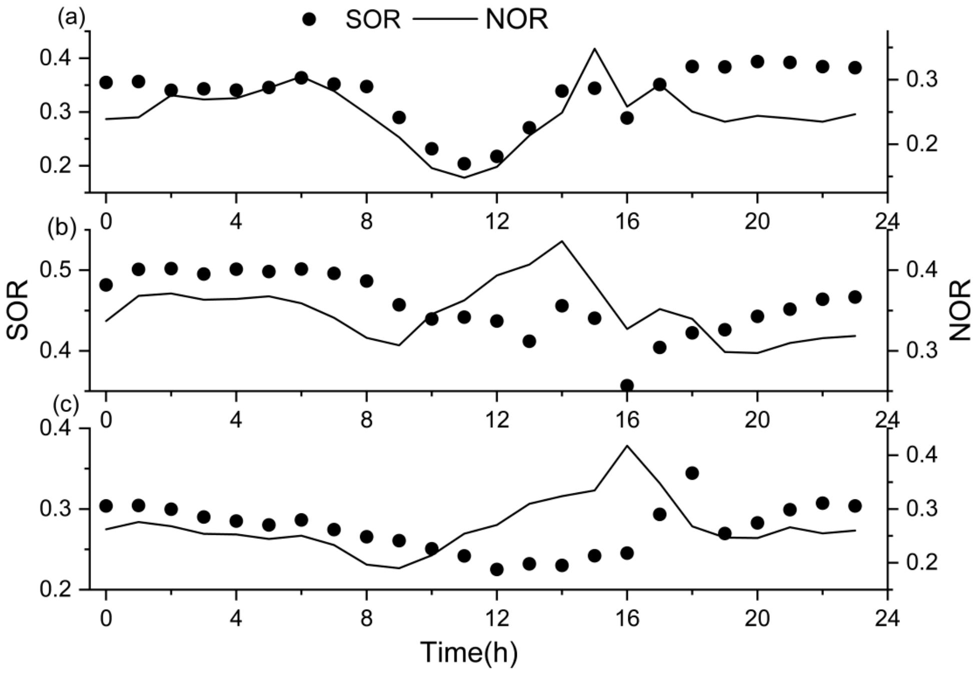

Figure S4. The diurnal variations of NOR and SOR in 2019 (a), 2020 (b) and 2021 (c).

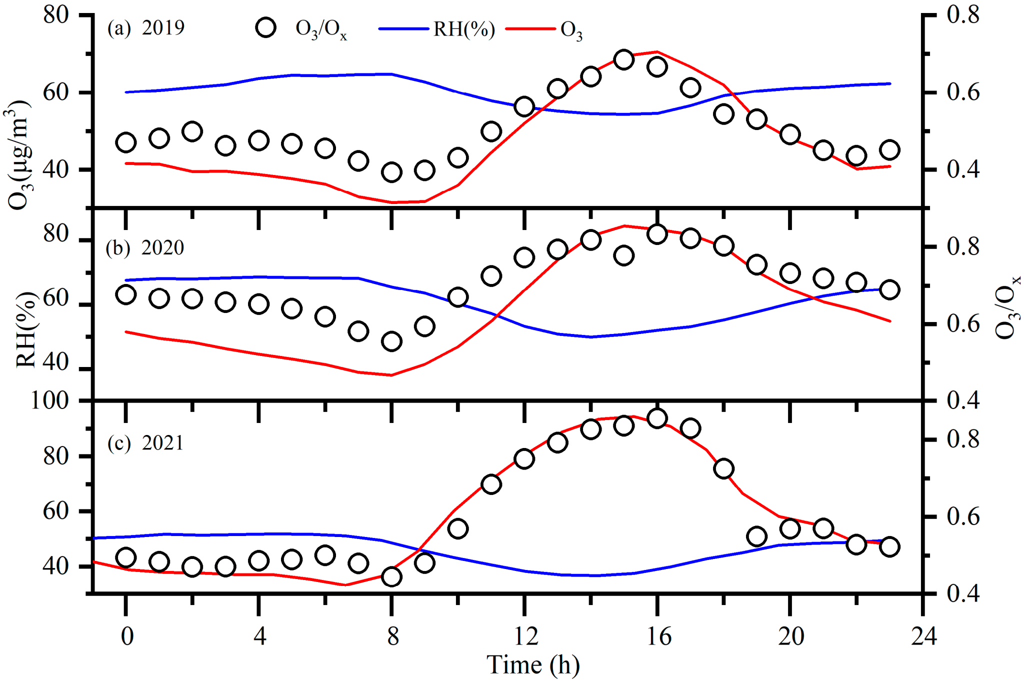

Figure S5. The diurnal variations of RH, O_3 and O_3/O_x in 2019 (a), 2020 (b) and 2021(c).

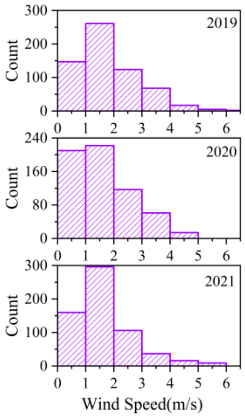

Figure S6. Statistical analysis of wind speed in 2019-2021.
